# Blocking IL-10 signalling at the time of immunization does not increase unwanted side effects in mice

**DOI:** 10.1186/s12865-017-0224-x

**Published:** 2017-08-15

**Authors:** Guoying Ni, Zaowen Liao, Shu Chen, Tianfang Wang, Jianwei Yuan, Xuan Pan, Kate Mounsey, Shelley Cavezza, Xiaosong Liu, Ming Q. Wei

**Affiliations:** 1 School of Medical Science, Griffith Health Institute, Griffith University, Gold Coast, QLD 4333 Australia; 2 Cancer Research Institute, The First People’s Hospital of Foshan, Foshan, Guangdong 528000 China; 3 Inflammation and Healing Research Cluster, University of the Sunshine Coast, Maroochydore DC, QLD 4558 Australia; 4 Molecular diagnosis and Target Therapy Laboratory, The First Affiliated Hospital of Guangdong Pharmaceutical University, Guangzhou, Guangdong China

**Keywords:** Il-10, CD4+ T cells, Intestine, Inflammation, Immunotherapy

## Abstract

**Background:**

Cancer therapeutic vaccine induced cytotoxic T cell (CTL) responses are pivotal for the killing of tumour cells. Blocking interleukin 10 (IL-10) signalling at the time of immunization increases vaccine induced CTL responses and improves prevention of tumour growth in animal models compared to immunization without an IL-10 signalling blockade. Therefore, this immunization strategy may have potential to curtail cancer in a clinical setting. However, IL-10 deficiency leads to autoimmune disease in the gut. Blocking IL-10 at the time of immunization may result in unwanted side effects, especially immune-pathological diseases in the intestine.

**Methods:**

We investigated whether blocking IL-10 at the time of immunization results in intestinal inflammation responses in a mouse TC-1 tumour model and in a NOD autoimmune disease prone mouse model.

**Results:**

We now show that blocking IL-10 at the time of immunization increases IL-10 production by CD4+ T cells in the spleen and draining lymph nodes, and does not result in blood cell infiltration to the intestines leading to intestinal pathological changes. Moreover, immunization with papillomavirus like particles combined with simultaneously blocking IL-10 signalling does not increase the incidence of autoimmune disease in Non-obese diabetic (NOD) mice.

**Conclusions:**

Our results indicate that immunization with an IL-10 inhibitor may facilitate the generation of safe, effective therapeutic vaccines against chronic viral infection and cancer.

**Electronic supplementary material:**

The online version of this article (doi:10.1186/s12865-017-0224-x) contains supplementary material, which is available to authorized users.

## Background

Cervical cancer is the second most common cancer in women worldwide [1, 2]. Chronic infection of human papillomavirus (HPV), especially HPV subtype 16 and 18, leads to the development of cervical cancer [3–6]. Although a prophylactic vaccine against HPV infection has been introduced, treatment of patients with chronic HPV infection and cervical cancer, remains a big challenge [7, 8].

Therapeutic vaccine targeting viral infected or cancer cells without hurting normal tissues or organs, is a potential therapeutic for the treatment of chronic HPV infection and HPV infection related cancers, by stimulating cytotoxic T cells (CTLs) against viral/tumour antigens [9]. The HPV early proteins E6 and E7 are expressed in HPV-associated cancers and are ideal targets for a therapeutic vaccine [10, 11].

Recently, much advance has been made in the area of HPV therapeutic vaccine research area [2, 12, 13]. Long E7 peptide/Incomplete Freunds adjuvant (IFA) and a nuclear acid vaccine (VGX-3100) have been shown to have therapeutic efficacy against HPV infection related pre-cancerous lesions [1]. However, up to now, HPV therapeutic vaccines are still not able to show efficacy against cervical cancer [14]. Current therapeutic vaccines often fail to generate enough numbers of CTLs, and as the tumour micro-environment is immunosuppressive, this prevents CTLs from killing tumour or viral infected cells [12]. To be more effective, therapeutic vaccines need to elicit the right type, and enough numbers of effector cells either through generation of new effector cells or through the activation of endogenous effector cells; these effector cells must then be able to migrate to tumour sites; overcome the immunosuppressive tumour micro-environment and finally kill the viral infected or tumour cells [15–19].

Interleukin 10 (IL-10) is an anti-inflammatory cytokine with the ability to suppress excessive inflammatory responses to both self and foreign antigens, through interacting with IL-10 receptors on the membranes of their target cells [20–22]. Cancer patients often have increased levels of serum IL-10; and increased levels of IL-10 often indicate poor prognosis [23–26]. IL-10 is detected in a variety of freshly excised human tumour samples [26–28]. IL-10 can be secreted by different types of cells, including tumour cells, and hematopoietic cells that infiltrate the tumour tissues. Tumour associated T regulatory cells and tumour associated antigen presentation cells, such as dendritic cells and macrophages, have been shown to produce IL-10 [27]. IL-10 secreting regulatory T cells inhibit vaccine induced CD8+ T cell responses, and can be amplified after therapeutic vaccination [26, 29].

Blocking IL-10 at the time of immunization drastically increases vaccine induced CTLs [30]. Recently, it has been demonstrated that blocking IL-10 concomitantly with immunization using Toll like receptor agonist inhibits tumour growth in a human papillomavirus E7 transformed TC-1 tumour mouse model, similar to a long E7 peptide/IFA vaccine that is effective against HPV infection related pre-cancer [11]. Moreover, it has been shown that immunization and simultaneous IL-10 signalling blockade better control tumour growth in a TC-1 mouse model than immunization without IL-10 signalling blockade [29]. Therefore, a therapeutic vaccine that contains IL-10 signalling inhibitor may be effective against cervical cancer. IL-10 signalling inhibitors have been developed for possible translation this novel immunization strategy into clinical practice [31, 32].

However, IL-10 deficiency in mice results in autoimmune disease in the intestinal tract driven by unimpeded reactivity of effector CD4+ T cells to antigens of intestinal microorganisms [33]. IL-10 deficiency increases hepatic damage, and leads to more severe disease during acute murine cytomegalovirus infection [34]. IL-10 is required for the generation of IL-10 secreting CD4+ T cells in vitro and in vivo [35]. Therefore, blocking IL-10 signalling at the time of immunization may also prevent the generation of antigen induced IL-10 secreting cells and result in unwanted side effects, especially in the intestine. Concerns about the development of autoimmune disease may prevent the inclusion of IL-10 inhibitor as a component of a therapeutic vaccine.

In the current paper, we investigated whether immunizing mice using TLR4 ligand as adjuvant and 2 different antigens (HPV16 E7 peptide, soluble antigen OVA), and simultaneously blocking IL-10 signalling would lead to inflammation in the gut and cause autoimmune disease. Unexpectedly, the numbers of IL-10 producing cells were significantly increased in mice immunized with HPV16 E7 peptide when IL-10 signalling is blocked compared to mice immunized without IL-10 signalling blockade.

## Methods

### Mice

Six to eigth weeks old, specific pathogen free (SPF) adult female C57BL/6 (H-2^b^) mice were ordered from the Animal Resource Centre, Sun Yat-Sen University and kept at the Animal Resource Centre, Sun Yat-Sen University, Guangdong province, China. Experiments in the current paper were approved by and then performed in compliance with the guidelines of Foshan First Peoples Hospital Animal Experimentation Ethics Committee (Ethics Approval Number: FSFH20160316). Mice were sacrificed by neck dislocation after the experiments. Mice were treated with dextran sulfate sodium sodium (DSS) inducing ulcerative colitis as mouse models of intestinal inflammation described elsewhere [30]. Six to eigth weeks old adult female autoimmune prone Non-obese diabetic (NOD)/Lt mice were purchased SPF from the Animal Resource Centre, Perth, Australia. NOD/Lt mice experiments were carried out at Princess Alexandra Hospital Animal House Facilities, University of Queensland. The experiment with NOD mice was approved by and performed in compliance with the guidelines of the University of Queensland Animal Experimentation Ethics Committee. Blood glucose levels of NOD/Lt mice were monitored weekly. Following two consecutive weekly blood glucose readings >12 mmol/l, mice were considered to be diabetic and were sacrificed. Mice were sacrificed by CO2 inhalation after the experiments.

### Peptides and antibodies

Anti-CD45.2-FITC (104); Anti-CD11b-PE (M1/70), Anti-CD4-FITC (RM4–4), anti-CD4-PE (RM 4–5), anti-GITR-PE (RAM34), anti-IL-10 (JES5-16E3), Anti-IFNγ-PE (XMG1.2), anti-IL-10-APC (JES5-16E3) were purchased from eBioscience (San Diego, CA, USA). Anti-CD3-PE (17A2), Anti-CD4-APC (RM4–5) mAbs were purchased from BioLegend (San Diego CA). Anti-mouse/Rat Foxp 3 staining kit was purchased from eBioscience (San Diego, CA, USA). Anti-CD3 mAb (CD 3–12) was purchased from GeneTex (Alton Parkway Irvine, CA, USA), anti-Ly-6G (1A8) was purchased from BioLegend (San Diego, CA, USA).

Ovalbumin (OVA), Lipopolysaccharide (LPS), Monophosphoryl Lipid A (MPLA) and Dextran Sulfate Sodium Salt (DSS) were bought from Sigma. Anti-IL10 receptor (1B1.3a) Monoclonal Antibody (MAb) was ordered from BioXcell, USA and stored at -80 °C till further use.

Long HPV16 E7 peptide GQAEPDRAHYNIVTFCCKCDSTLRLCVQSTHVDIR, HPV16 E7 the MHC class I (H-2 Db) restricted epitope RAHYNIVTF, the MHC class I (H-2 D^b^) restricted ovalbumin (OVA) peptide SIIFINKLE, and the MHC class II (H-2 Db) restricted peptide ISQAVHAAHAEINEAGR were synthesised and purified by Mimotopes (Melbourne, Australia). The purity of the synthesised peptides were 95% and was determined by reverse-phase HPLC. Peptides were dissolved in 0.5% DMSO in PBS and stored at −20 °C till use.

### Production of recombinant VLPs

Papillomavirus virus like particles (VLPs) L1E7 were produced, purified and confirmed as described elsewhere [30, 36].

#### 2.1.4 immunization of mice

Groups of three to six mice were immunized as indicated with a): 50 μg of OVA and 15 μg of LPS subcutaneously (s.c.). with or without Intraperitoneal injection (i.p.) of 500 μg of anti-IL10R antibodies; or b): with 50 μg of long E7 peptide/15 μg of Monophosphoryl Lipid A (MPLA) with 300 μg of anti-IL10R antibodies or control antibodies s.c; or c): with 50 μg of VLPs intramuscularly (i.m.), with or without i.p. injection of 500 μg of anti-IL10R antibodies at 14 days apart.

### ELISA for IL10 and IFN-γ cytokines from culture supernatants

ELISA for IL-10 and IFN-γ (ebioscience, USA) was performed according to the manufacturer’s recommended procedures and were described elsewhere [37].

#### ELISPOT

ELISPOT for antigen specific IFNγ CD8 T cell response was performed as previously described [37]. Briefly, single spleen cell suspensions were added to the 96 well plates (Millipore, Bedford, MA) previously coated with anti-IFN-γ (BD Harlingen, San Diego, CA). RAHYNIVTF was added and cells cultured at 37 °C overnight. Biotinylated anti-IFN-γ (BD Harlingen), avidin –horseradish peroxidase (Sigma-Aldrich) were sequentially added before developed with DAB (Sigma-Aldrich). Experiment was stopped by washing with tap water. The results were determined by an ELISPOT reader (AID Autoimmun Diagnosticka GmbH, Strassberg, Germany).

### Intracellular staining for IFNγ, IL-10

Single spleen cell suspensions or single lymph node cell suspension obtained from immunized and control mice were stimulated with PMA and ionomycin for 3–5 h in the presence of protein transport inhibitor monensin (BioLegend). For some experiments, the cells were cultured for 72 h in the presence of 1 μg/ml of OVA, 1 μg/ml of SIINFEKLE or 1 μg/ml of ISQAVHAAHAEINEAGR and then stimulated with PMA and ionomycin for 3–5 h. After specific or non-specific stimulation, the cells were firstly surface stained with anti-CD3, CD4 or CD8 antibodies before intracellularly stained for IL-10 and IFN-γ, by using commercial Per/Fix reagents (BD Pharmingen) [37].

### Isolation and staining of intestinal lymphocytes (IELs)

The intestinal lymphocytes (IELs) were isolated by following Lefrancois’s protocol [38]. Briefly, the mice were sacrificed by cervical dislocation and the intestines were harvested and separated from unwanted fat and connective tissue. Fecal matter in the intestinal was expelled by flushing the entire intestine with 40 ml, 4 °C CMF solution. After removing remaining fat and connective tissues and Peyer’s patches, the intestines were then cut longitudinally and laterally into ~ 0.5 cm pieces. The intestine pieces were washed with 4 °C CMF solution until supernatants were relatively clear. The intestinal pieces were incubated with 20 ml CMF/FBS/DTE solution at 37 °C with stirring for another 20 min. The intestinal pieces were then transferred to a 50-ml conical centrifuge tube and vortex for 15 s at maximum setting. Intestinal pieces were allowed to settle and supernatant transferred to another 50-ml conical tube, followed by a 10-min incubation on ice. Supernatants contained the IEL and some epithelial cells. The IEL supernatant was then centrifuged at 350 g for 5 min and the supernatant discarded. Counted viable cells were re-suspended in Cell Staining Buffer at 5 × 10^6^ cells/ml. Fc receptors were then blocked with 1.0 μg Anti-Mouse CD16/CD32 Purified (clone: 93) for 10 min on ice, then anti-mouse CD45.2 FITC, anti-mouse CD3 PE, anti-mouse CD8 PerCP Cyanine5.5 and anti-mouse CD4 APC added as appropriate and incubated on ice for 20 min in the dark. The cells were acquired on BD FACSCalibur. Flow cytometry data was analyzed by flowjo.

### Immunohistochemistry (IHC) for the detection of intestinal infiltrating CD3^+^ T cells and neutrophils

Intestines from immunized and control mice were excised and cut into pieces as described above. Mouse intestine pieces were fixed in 10% buffered neutral formalin, then processed through graded ethanol and three changes of xylene, before infiltrated with paraffin. Samples were embedded into paraffin blocks and cut into 4 mm sections before mounted onto slides. The Vectastain® ABC kit was used for antibody detection and the methods has been described elsewhere [31]. Briefly, Anti-CD3 mAb (clone CD3–12) or anti-Ly-6G (clone 1A8) were used to detect T cells and neutrophils. Murine lymph node was used as the positive control for CD3+ T cells staining. Slides from the same series without primary antibody were used as negative controls.

CD3 positive or anti-Ly-6G positive cells were quantified using Image analysis software Image pro Plus 6.0 (IPP). The infiltrating CD3+ T cells or neutrophils was quantified from five high-power fields (HPFs, 200×) and intestinal infiltrating CD3+ T cell or neutrophil numbers were calculated as the ratio of integrated option density IOD /area (IOD/area) [39].

### Statistical analysis

Statistical analysis was performed by using the two tailed Student’s test, or Log rank test using Prism 4.0 (Graphpad Software, San Diego).

## Results

### Blocking IL10 signalling at the time of immunization increases the numbers of IL-10 producing cells

**Fig. 1:**
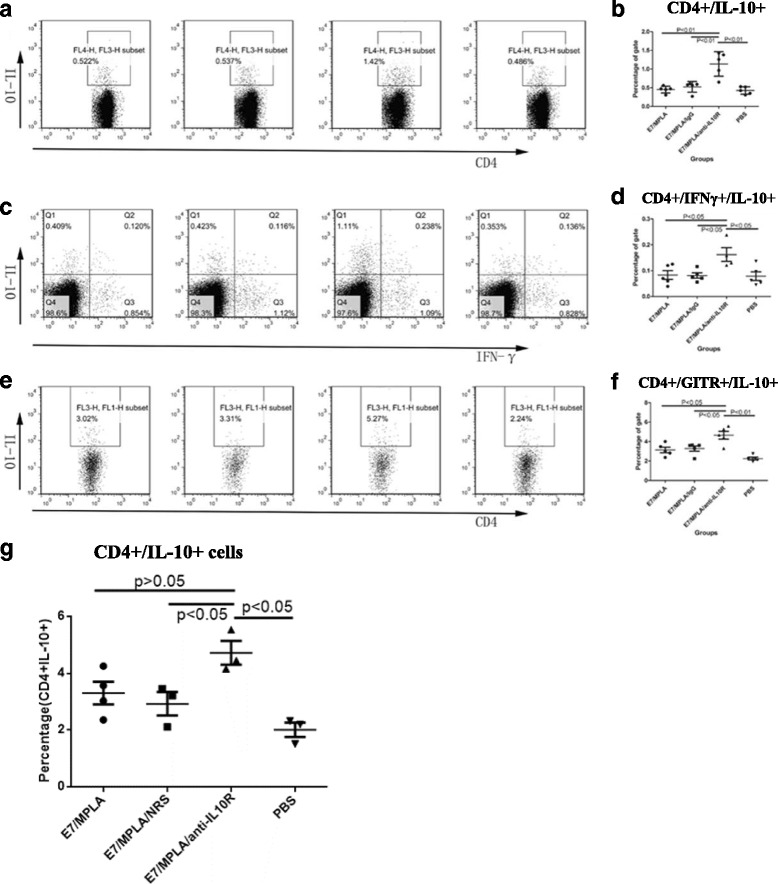
Blocking IL-10 signalling at the time of long HPV16 E7 peptide/MPLA immunization increases the numbers of IL-10 producing cells. Groups of five C57BL/6 mice were immunized as indicated with 50 μg of E7 peptide/15 μg of Monophosphoryl Lipid A (MPLA), 300 μg of anti-IL10R antibodies or control antibodies s.c on days 0 and 14. Splenocytes and draining lymph node cells from immunized mice were harvested and stimulated with 25 ng/ml PMA and 1 μg/ml ionomycin for 6 h in the presence of monensin on 7 days after final immunization. Cells were surface stained for CD3, CD4, and GITR and intracellular stained for IL-10 and IFN-γ. CD3+ cells were gated. Results for cells of draining lymph nodes were shown. A and B: IL-10 secreting CD3 + CD4+ T cells IL-10 (**a**) FACS plot and (**b**) summarised data showing IL-10 expression by CD3 + CD4+ T cells. C and D: CD3 + CD4+ T cells secreting IL-10 and IFN-γ *dot* plots (**c**) and summarised data from different groups (**d**) E and F: CD4 + GITR+ T cells secreting IL-10: FACS profile (**e**) and summarised data from different groups (**f**). Splenic CD4 + IL-10+ cells were shown in (**g**)

We have previously shown that HPV16 early protein E7 long peptide/MPLA immunization and simultaneously blocking IL-10 signalling enhances vaccine induced E7 specific CD8+ T cell responses compared with immunization without IL-10 signalling blockade [11]. We investigated the number of IL-10 secreting CD4 + T cells in HPV16 early protein E7 long peptide/MPLA immunized mice with or without blocking IL-10 signalling. Mice were immunized twice subcutaneously at 2 weeks apart, with long E7 peptide/MPLA with or without administration of anti-IL-10R antibodies subcutaneously. Splenocytes and lymphocytes from draining lymph nodes were stained for CD3, CD4, GITR and IL-10. While the total numbers of splenic cells and draining lymph nodes were increased in IL-10 signalling blocked group, the ratio of CD4+ and CD4 + GITR+ T cells were slightly increased in IL-10 signalling blocked group compared with non-IL-10 signalling blocked group (data not shown). Unexpectedly, the numbers of IL-10 producing CD4+ T cells were significantly increased in the long E7/MPLA immunized and simultaneously IL-10 signalling blocked mice compared to mice immunized without IL-10 signalling blockade (Fig. 1a, b). The numbers of CD4 + GITR+ IL10+ T cells were also significantly increased in IL-10 signalling blocked and long E7 peptide/MPLA immunized mice (Fig. 1e, f) as measured by IL-10 intracellular staining in CD4 + GITR+ T cells. The numbers of IFNγ + IL10 + CD4+ T cells that limit the strength of Th1 responses [38] were also increased in immunized and IL-10 signalling blocked mice (Fig. 1c, d). The numbers of CD4 + IL-10+ cells in the spleen of IL-10 signalling blocked group is also increased compared with IL-10 non-blocked group (Fig. 1g).

We further confirmed this phenomenon by using another antigen ovalbumin (OVA). Similar to papillomavirus (PV) virus like particles (VLPs) and HPV16E7 long peptide immunization, blocking IL-10 with simultaneous OVA/LPS immunization enhanced the vaccine induced antigen specific CD8+ T cell response by ELISPOT, intracellular staining and ELISA (Additional file 1: Figure S1a). To investigate if the numbers of IL-10 secreting CD4+ T cells were increased after OVA/LPS immunization and IL-10 signalling blockade, mice were immunized with OVA/LPS, with or without administration of anti-IL-10R antibodies twice, splenocytes and lymphocytes isolated from draining lymph nodes were stained for IL-10. Similarly, splenic IL-10 secreting CD4+ T cells, CD4 + GITR+ IL10+ T cells, and CD4 + IFNγ + IL-10+ T cells were significantly increased in mice immunized with OVA/LPS plus administration of anti-IL-10R antibodies, compared with mice immunized with OVA/LPS without administration of anti-IL-10R antibodies (Additional file 1: Figure S2).

### Blocking IL10 signalling at the time of immunization in mice does not increase the numbers of spleen CD4 + Foxp3+ T cells

**Fig. 2:**
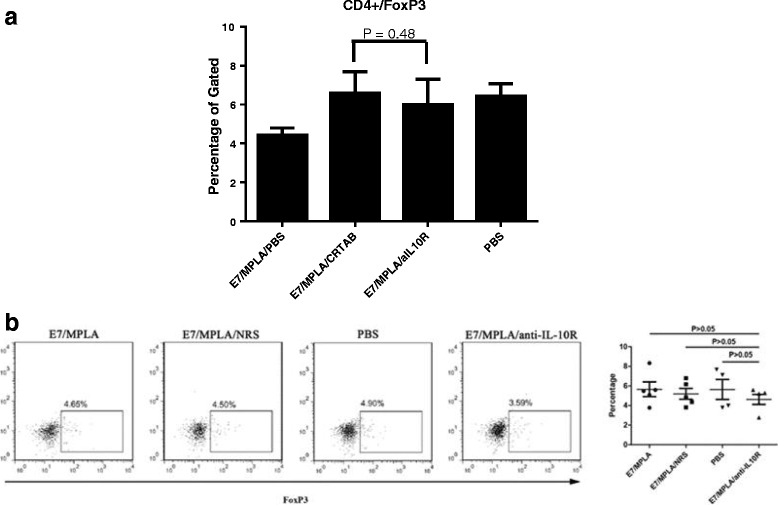
Blocking IL-10 signalling at the time of immunization does not increase the numbers of CD4 + Foxp3+ T cells. Group of 4 C57BL/6 mice were immunized with 50 μg of long E7 peptide/10 μg of MPLA, 50 μg of long E7 peptide/10 μg of MPLA/300 μg of anti-IL10R antibodies, 50 μg of long E7 peptide/10 μg of MPLA/300 μg of Normal Rat Serum or PBS twice respectively at 14 days apart subcutaneously, 1 weeks after final immunization; splenocytes and lymphocytes from draining lymph nodes were collected and single cells made; cells were stained for CD3, CD4 and intracellularly stained for Foxp3 as described in Methods. CD3+ cells were gated. Figure shows the numbers of CD4 + Foxp3+ cells in draining lymph nodes of different immunization groups in the draining lymph nodes (**a**) and spleen (**b**)

We next investigated whether the numbers of Foxp3 + CD4+ T cells are changed after blocking IL-10 signalling at the time of immunization. Mice were immunized with HPV16E7 peptide/MPLA in the presence or absence of anti-IL10R antibodies, and the numbers of CD4 + Foxp3+ T cells from spleen and draining lymph nodes were measured by flow cytometry. The results showed that the numbers of CD4 + Foxp3+ T cells were similar in the draining lymph nodes (Fig. 2a) and spleen (Fig. 2b), whether the mice were immunized simultaneously with or without anti-IL10R antibody administration. Similar results were obtained in mice immunized with OVA/LPS in the presence or absence of anti-IL-10R antibodies (Additional file 1: Figure S3).

### Neutralizing IL10 at the time of immunization does not cause pathological changes in intestines

**Fig. 3:**
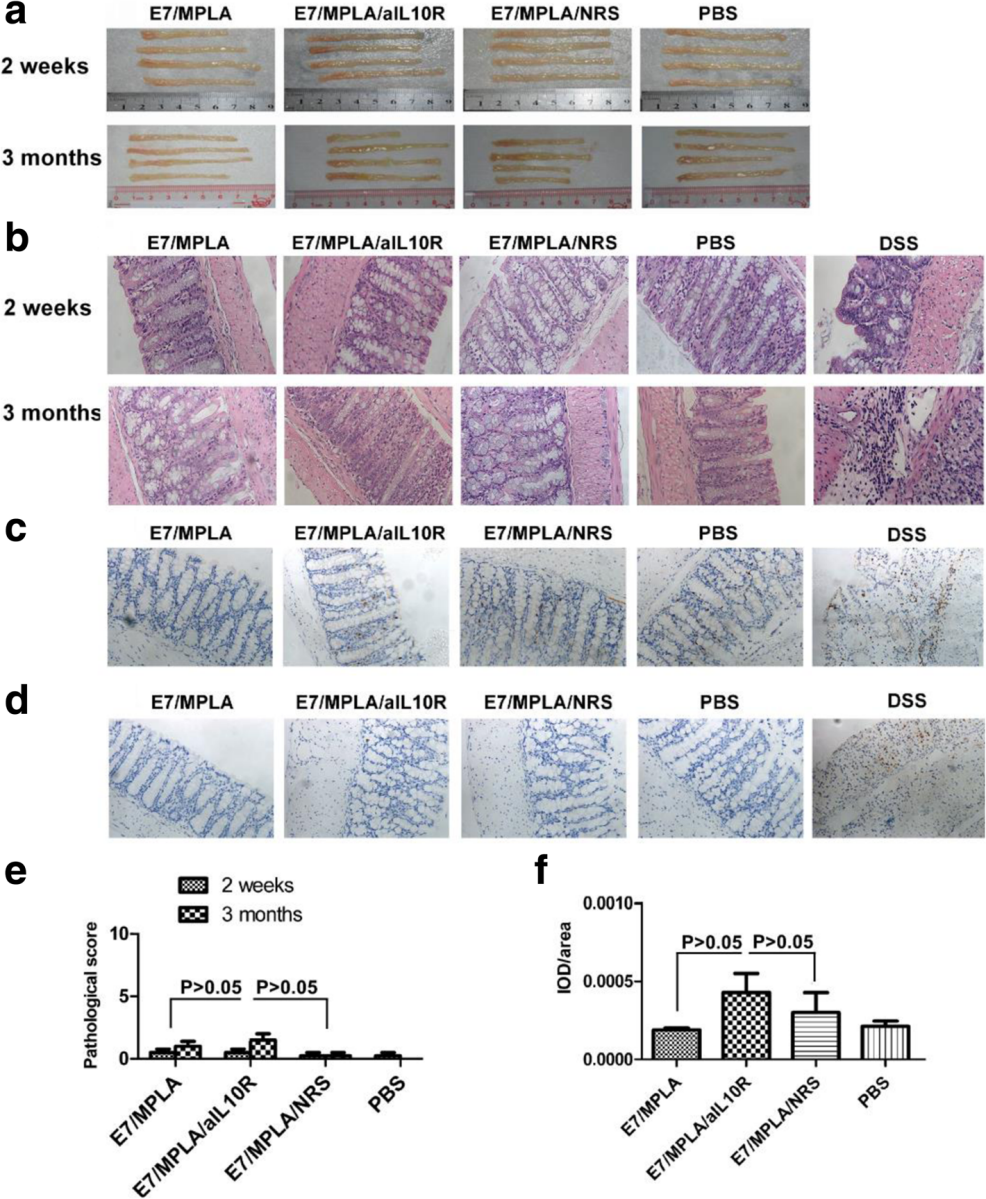
Neutralizing IL-10 at the time of immunization does not cause inflammation in intestines. Group of 4 C57BL/6 mice were immunized with 50 μg of long E7 peptide/10 μg of MPLA, 50 μg of long E7 peptide/10 μg of MPLA/300 μg of anti-IL10R antibodies, 50 μg of long E7 peptide/10 μg of MPLA/300 μg of Normal Rat Serum or PBS twice at 14 days apart subcutaneously, 2 weeks or 3 months after final immunization. Mouse model of ulcerative colitis was induced by administering 5% DSS in the drinking water for 10 days. Samples of intestines were collected and examined for blood cells infiltration. **a**: Morphology of intestinal tract. **b**: Histology characterization of intestinal tract. Intestinal tract section were stained with hematoxylin/eosion (200 × magnification).**c, d**: Immunohistochemistry characterization of intestinal tract 2 weeks after final immunization. Intestinal tract section were incubated with anti- CD3 (**c**) or anti-Ly-6G mAb (**d**). **e**: Pathological score of intestinal tract. **f**: Expression of CD3 was calculated as integrated option density IOD /area (IOD/area) 2 weeks after final immunization. Results are from pooled two independent experiments (**e, f**)

We investigated whether blocking IL-10 signalling at the time of immunization causes inflammation in important tissues and organs. We especially wished to know whether this immunization strategy causes inflammation in the intestine, as IL-10 knockout mice have chronic inflammation in their intestine late in their life. Mice were immunized with long E7 peptide/MPLA twice at 14 days apart, in the presence or absence of anti-IL10R administration. 2 weeks after the final immunization there were no signs of inflammation observed in the heart, brain, kidney, and intestine by eye or under the microscope (data not shown and Fig. 3a) in mice immunized with long E7 peptide/MPLA, long E7 peptide/MPLA/anti-IL10R antibody, long E7 peptide/MPLA/Normal Rat Serum or PBS, while mice with DSS-induced ulcerative colitis had significant infiltration of blood cells in intestine (Fig. 3). Pathological score of intestinal inflammation was determined by a pathologist blinded to which immunization group the mouse was from, using a method published elsewhere [40]. The pathological score was also similar among different immunization groups (Fig. 3). Furthermore, intestinal samples were stained with anti-CD3 (Fig. 3), or with anti-Ly-6G (1A8) (Fig. 3) for T cells and neutrophils; both T cell and neutrophil infiltration were similar among different immunization groups (Fig. 3).

**Fig. 4:**
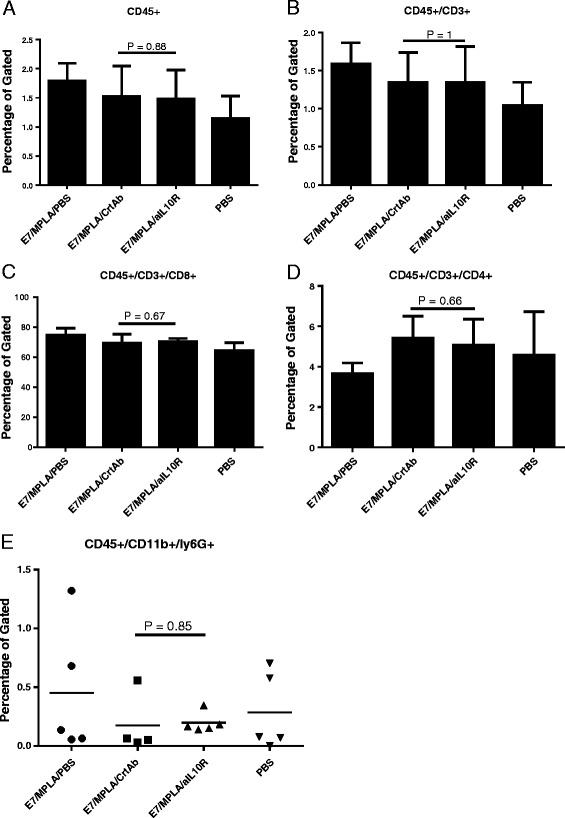
Neutralizing IL-10 at the time of immunization does not attract T cells and neutrophils to the intestines of immunized mice**.** Group of 4 C57BL/6 mice were immunized with 50 μg of long E7 peptide/10 μg of MPLA, 50 μg of long E7 peptide/10 μg of MPLA/300 μg of anti-IL10R antibodies, 50 μg of long E7 peptide/10 μg of MPLA/300 μg of Normal Rat Serum or PBS twice at 14 days apart subcutaneously. 1 weeks after final immunization; samples of intestines were collected and examined for blood cells infiltration as described in Methods. CD45+ cells were gated. **a**: Total CD45+ cells, **b**: CD3+ T cells, **c**: CD8+ T cells, **d**: CD4+ T cells, **e**: CD45 + CD11b + Ly6G+ cells

In another experiment intestines from different immunization and control groups, 1 week after final immunization, were cut into pieces and IELs isolated following the procedures described above. The IELs were stained for CD45+ cells, T cells and neutrophils and results were analysed by flow cytometry. The results showed that the numbers of total CD45+ cells, and the numbers of both T cells and neutrophils were similar between mice immunized with long E7 peptide/MPLA, with or without blocking IL-10 with anti-IL-10R antibodies (Fig. 4).

### Neutralizing IL10 at the time of immunization does not increase the incidence of diabetics in NOD mice

**Fig. 5:**
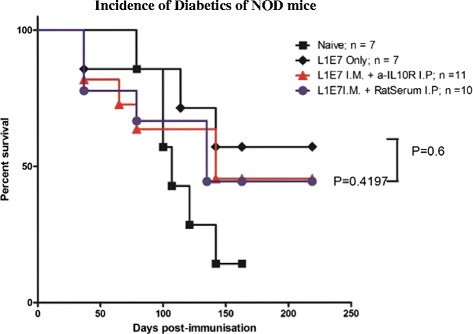
Neutralizing IL-10 at the time of immunization does not increase the incidence of diabetics in NOD mice. 6–8 weeks old adult female autoimmune prone NOD/Lt mice were divided into different groups. No treatment group (*square*), VLP immunization (*Diamond*), VLP immunization plus Normal Rat Serum (*Down Triangle*), VLP immunization plus anti-IL10R antibody (*Cycle*). Mice were either untreated or immunized twice with 50 μg of papillomvirus like particles with or without 500 μg of anti-IL10R antibody *i.p.* on day 0 and 14. The development of diabetes was monitored weekly. Results are shown of pooled results from two independent experiments

We showed that blocking IL-10 at the time of immunization increases vaccine induced CTL responses, whether the antigens is papillomavirus like particles [41]; soluble protein [42] or peptide [40]. As we are interested in applying this novel immunization strategy to clinic, papillomavirus like particles are licensed in 2006, next we investigated whether autoimmune prone NOD mice were more likely to develop diabetes when IL10 signalling was blocked at the time of immunization. NOD mice, which develop diabetes spontaneously, were immunized twice using papillomavirus like particles 14 days apart, with or without neutralizing IL-10 antibody. The incidence of diabetes was similar amongst the immunized, immunized/anti-IL10R antibody, and immunized/Normal Rat Serum (NRS) groups (*p > 0.05*, Log rank test). These results suggest that temporarily blocking IL-10 at the time of immunization is safe, and does not increase the incidence of autoimmune disease in autoimmune prone mice (Fig. 5).

## Discussion

In the current study, we demonstrated that temporal blocking of IL-10 signalling at the time of immunization does not result in the infiltration of T cells and neutrophils to the intestine. This immunization strategy also does not increases the development of autoimmune disease in NOD mice. We also showed by using two different antigens, OVA and HPV16E7 long peptide as the immunogen, that the numbers of IL-10 producing CD4+ T cells, including CD4 + GITR+ IL10+ cells and CD4 + IL10 + IFNγ + cells are significantly increased in mice immunized with OVA or E7 long peptide simultaneously with IL-10 signalling blockade, than those immunized without IL-10 signalling blockade.

IL-10 signalling blockade at the time of immunization increases vaccine induced cytotoxic T cell responses [9, 43]. The increased CD8+ T cell responses by IL-10 signalling blockade can be achieved by the administration of anti-IL10R antibodies subcutaneously and with different TLRs and Incomplete Freund Adjuvant (IFA) [9]. In a mouse chronic LCMV infection model, blockade of IL10 increases the efficacy of a therapeutic DNA vaccine by increasing vaccine induced T cell responses and enhancing the clearance of persistent LCMV replication [44–46]. Intra-tumour injection of Toll like receptor 9 ligand CpG, plus anti-IL-10 receptor antibody intraperitoneally, leads to tumour rejection in mouse tumour models [47]. Recently, it was shown in a human papillomavirus 16 tumour antigen transformed TC-1 tumour model that immunization plus IL10 signalling blockade prevents TC-1 tumour growth [11], and moreover, this immunization strategy improved the prevention of tumour growth than immunization without IL-10 signalling blockade [29]. Blocking IL-10 at the time of immunization also promotes the generation of more IFNγ producing CD4+ T cells (Fig. S1). Therefore, therapeutic vaccines containing a IL-10 signalling inhibitor may be effective against chronic HPV infection and HPV infection related cancers in human.

IL-10 deficiency leads to autoimmune diseases in the gut [33]. IL-10 deficiency also increases hepatic immunopathology, leading to more severe disease and weight loss during acute murine cytomegalovirus infection [34]. Leishmania major infected IL-10−/− mice developed larger lesions but had fewer parasites due to the increased level of IL-17 [48]. Blocking IL-10 signalling at the time of immunization may therefore have the possibility of inducing autoimmune diseases in immunized patients. Interestingly, our results in NOD mice show that immunization with papillomavirus like particles, a TLR4 receptor stimulator, plus blocking IL-10 signalling does not increase the incidence of diabetes in NOD mice, suggesting this immunization strategy may be safe, as IL-10 signalling is blocked only for a short period. In support of the NOD mice results, we observed that blocking IL-10 signalling at the time of immunization in C57/BL6 mice induced mores IL-10 secreting CD4+ T cells than immunization without blocking IL-10 signalling. Antigen experienced CD4 + GITR + IL10+, CD4 + IL10 + IFNγ + cells but not CD4 + Foxp3+ T cells (Figs. 1, 2 and Fig. 3) were increased in immunized and IL-10 signalling blocked mice. CD4 + IL-10 + IFNγ + cells are a group of cells which control the Th1 responses. These IL-10 secreting Th1 CD4+ T cells have been identified in parasite infection models [49], with both effector and regulatory functions [50]. Factors influencing IL-10 secretion by IFNγ producing CD4+ T cells include antigen concentration, IL-12 and IL-27 levels, and expression of Notch and inducible costimulatory molecule ligands (ICOS-L) on dendritic cells [50]. The increased numbers of CD4 + GITR + IL10+ T cells and CD4 + IFNγ + IL10+ T cells in immunized and IL-10 signalling blocked mice indicates that the increased IFNγ secreting CD8 + T cell and CD4+ T cell responses are balanced with the increased numbers of IL-10 secreting T cells after vaccination and simultaneous blocking IL-10 signalling. Increased IL-10 levels secreted by CD4+ T cells in vaccinated and IL-10 signalling blocked mice may be beneficial to patients, as increased IL-10 may protect the patients from over reaction to the vaccination. Stronger T cell responses without IL-10 may induce autoimmune diseases in vaccinated patients.

## Conclusion

In summary, blocking IL-10 signalling at the time of immunization increases the numbers of IL-10 producing T cells; does not induce unwanted side effects, especially in the intestine of the immunized mice therefore this immunisation strategy may be effective against HPV chronic infection and HPV infection related cancers, with minimal chance of increasing the incidence of autoimmune diseases of immunized patients.

## Additional file

Additional file 1: Figure S1.
**S2** and **S3** Blocking IL-10 at the time of immunization with OVA increases vaccine induced antigen specific CD8+ and CD4+ IFNγ and IL-10 secreting CD4+ T cell responses but does not increase the numbers of CD4 + Foxp3+ T cells. (PDF 547 kb)

## Abbreviations

CTL: Cytotoxic T lymphocyte
HPV: Human papillomavirus
IFA: Incomplete Freunds adjuvant
IL-10: Interleukin 10
LPS: Lipopolysaccharide
MPLA: Monophosphoryl Lipid A
NOD: Non-obese diabetic
OVA: Ovalbumin
TEM: Transmission Electron Microscope
VLPs: Virus like particles
