# Supplementary material for: Blocking IL-10 signalling at the time of immunization does not increase unwanted side effects in mice
Source: BMC Immunol. 2017 Aug 15;18:40. doi: 10.1186/s12865-017-0224-x (PMC5557397; doi:10.1186/s12865-017-0224-x)
Supplement: Additional file 1: Figure S1. — S2 and S3 Blocking IL-10 at the time of immunization with OVA increases vaccine induced antigen specific CD8+ and CD4+ IFNγ and IL-10 secreting CD4+ T cell responses but does not increase the numbers of CD4 + Foxp3+ T cells. (PDF 547 kb) [file 12865_2017_224_MOESM1_ESM.pdf]

Supplementary Figure legend.

Figure S1 **Blocking IL-10 signalling at the time of immunization increases both antigen specific IFNγ secreting CD8+ T and CD4+ T cells responses**

Group of 3 C57BL/6 mice were primed with 50 µg of OVA, 15 µg of LPS at 14 days apart with or without 500 µg of anti IL10R Ab or normal rat serum (NRS). Six days after final examination, spleen cells from immunized mice were collected. ELISA, ELISPOT assay and Intracellular Staining for IFNγ was performed as described in materials and methods. A: Left: ELISPOT assay for CD8+ T cell responses to a MHC I restricted peptide SIIFNKEL.Middle: Percentage of CD8+ IFNγ+ T cells by flow cytometry analysis. Splenic cells pulsed with SIINFKEL overnight, and stained with CD4, CD3 and IFNγ. CD3+, CD4-. CD3+ cells were gated and CD3+CD4-IFNγ+ cells are shown. Right: Splenic cells pulsed with SIINFKEL overnight, supernatants were measured for the presence of IFNγ by ELISA. Results throughout are the mean±1SEM of three individual mice and represent one of two independent experiments. B: Splenic cells pulsed with OVA overnight, and stained with CD4, CD3 and IFNγ. CD3+ cells were gated and CD3+CD4+IFNγ+ cells are shown. Left: FACS profile; Right: Percentage of CD4+IFNγ+ T cells.

Figure S2. Blocking IL-10 signalling at the time of OVA/LPS immunization increases the numbers of IL-10 producing cells.

Group of 3 C57BL/6 mice were immunized with 50 µg of OVA and 15 µg of LPS with or without 500 µg of anti IL10R Ab twice at 14 days apart. Six days after final

immunization, spleen cells from immunized mice were collected for surface staining and intracellular staining for IFNγ and IL10. A: Splenic cells were collected and pulsed with 1μg/ml of OVA overnight, supernatants were measured for IL-10 by ELISA (Left). Or CD3+ lymphocytes were gated. CD4 and IL10 were stained as described in materials and methods. B: CD4, GITR and IL10 were stained. Left: FACS profile of CD4+GITR+IL-10+ cells, Right: summarised data of CD4+GITR+IL10+ cells. C: splenic cells were cultured overnight in the presence of 1μg/ml of OVA; cells were stained for CD4, IL10 and IFNγ. Left: FACS profile. Right: CD4+INFγ+IL10+ cells.Results throughout are the mean and 1 SEM of three individual mice and represent one of two independent experiments.

Figure S3 **Blocking IL-10 signalling at the time of OVA/LPS immunization in mice does not**

increases the numbers of CD4+Foxp3+ T cells.

Group of 3 C57BL/6 mice were immunized with 50 μg of OVA and 15 μg of LPS with or without 500 μg of anti IL10R Ab twice at 14 days apart. Six days after final immunization, spleen cells from immunized mice were collected for surface staining and intracellular staining of CD4 and Foxp3.

Figure S1

A: CD8+ cells

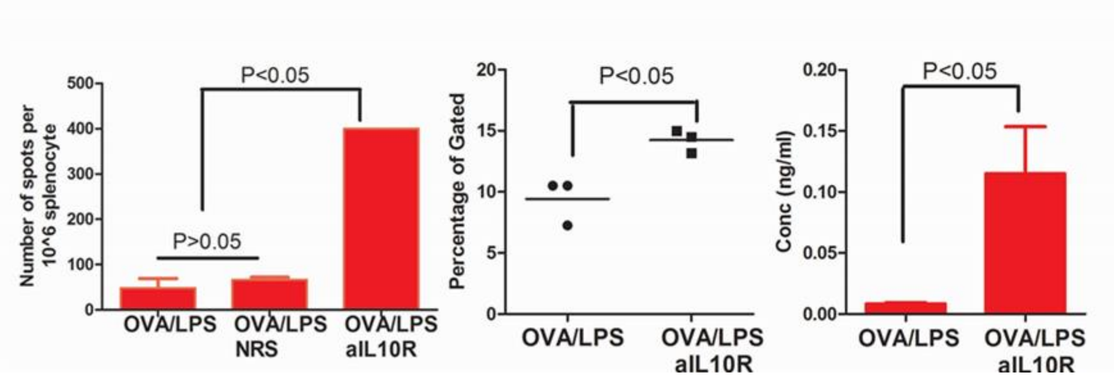

B: CD4+ cells

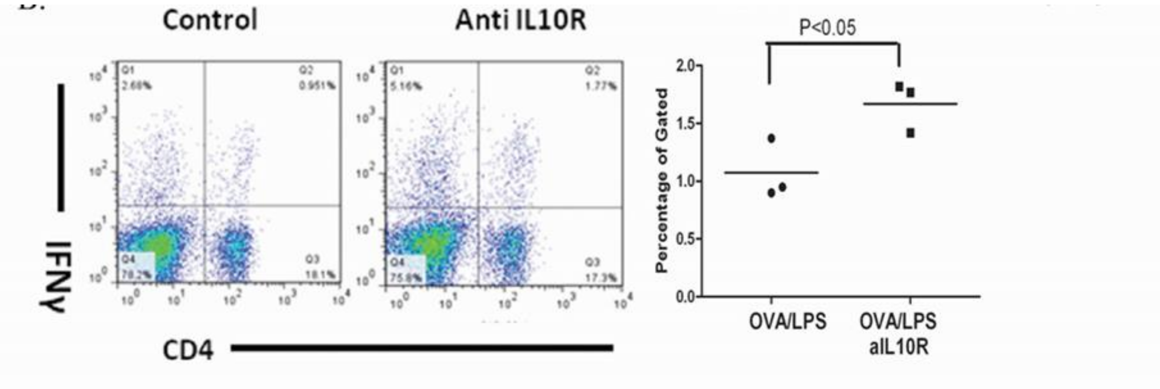

Figure S2

A

CD4+/IL-10+

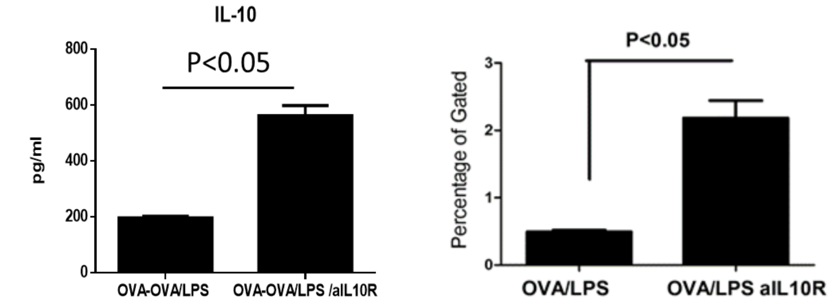

B

CD4+/GITR+/IL-10+

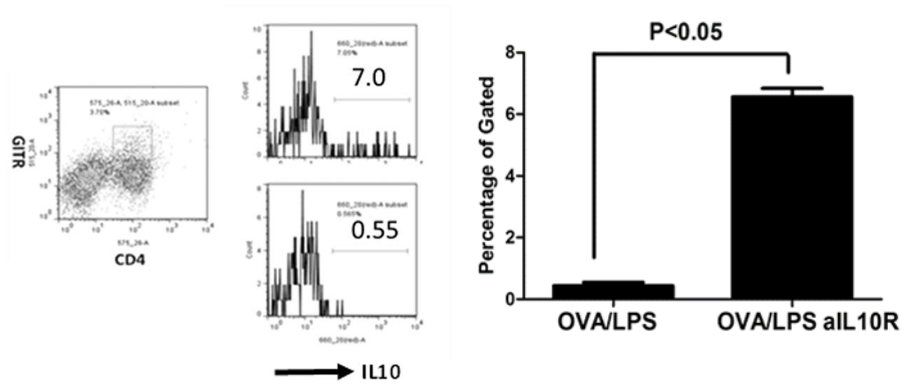

C

CD4+/IFNγ+/IL-10+

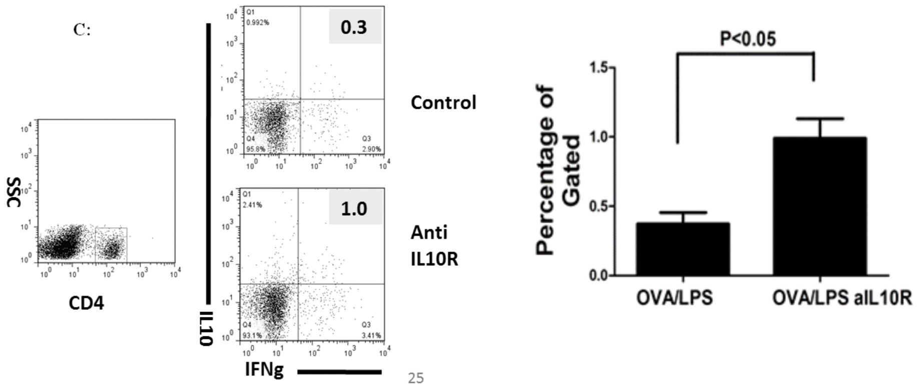

Figure S3

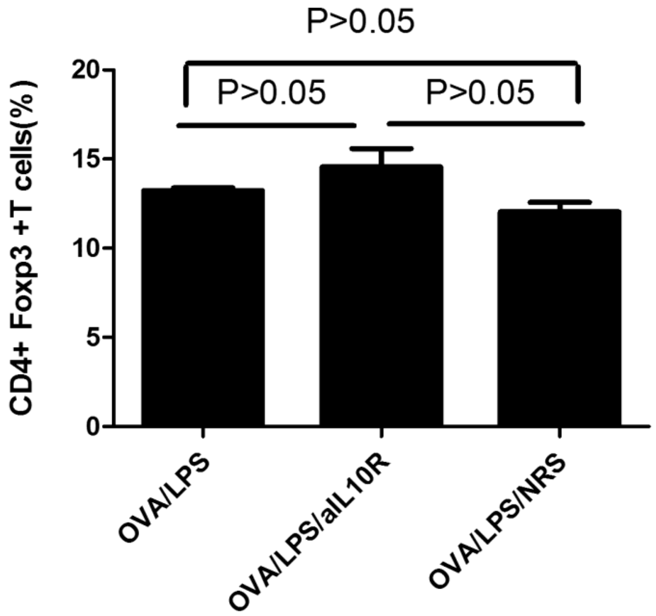
P>0.05
P>0.05
P>0.05
CD4+ Foxp3 +T cells(%)
20
15
10
5
0
OVA/LPS
OVA/LPS/aIL10R
OVA/LPS/NRS
